# Single dental implant retained mandibular complete dentures – influence of the loading protocol: study protocol for a randomized controlled trial

**DOI:** 10.1186/1745-6215-15-186

**Published:** 2014-05-24

**Authors:** Nicole Passia, Miha Brezavšček, Elfriede Fritzer, Stefanie Kappel, Thomas Kern, Ralph G Luthardt, Nadine Frfr von Maltzahn, Torsten Mundt, Michael Rädel, Axel von Stein-Lausnitz, Matthias Kern

**Affiliations:** 1 Department of Prosthodontics, Propaedeutics and Dental Materials, Christian-Albrechts University of Kiel, Kiel, Germany; 2 Department of Prosthetic Dentistry, University Hospital Freiburg, Freiburg, Germany; 3 Center for Clinical Studies, Christian-Albrechts University of Kiel, Kiel, Germany; 4 Department of Prosthodontics, Heidelberg University Hospital, Heidelberg, Germany; 5 Department of Prosthodontics and Biomaterials, University Hospital Aachen, Aachen, Germany; 6 Center of Dentistry, Department of Prosthetic Dentistry, Ulm University Hospital, Ulm, Germany; 7 Department of Prosthodontics, University Hospital Hannover, Hannover, Germany; 8 Department of Prosthetic Dentistry, Ernst-Moritz-Arndt University of Greifswald, Greifswald, Germany; 9 Department of Prosthetic Dentistry, Dresden University of Technology, Medical Faculty Carl Gustav Carus, Dresden, Germany; 10 Center for Dental and Oral Medicine, Department of Dental Prosthetics, University Medical Center Hamburg Eppendorf, Hamburg, Germany

**Keywords:** Single midline implant, Edentulous mandible, Mandibular complete denture, Implant-supported overdenture, Implant therapy

## Abstract

**Background:**

Over the years, there has been a strong consensus in dentistry that at least two implants are required to retain a complete mandibular denture. It has been shown in several clinical trials that one single median implant can retain a mandibular overdenture sufficiently well for up to 5 years without implant failures, when delayed loading was used. However, other trials have reported conflicting results with in part considerable failure rates when immediate loading was applied. Therefore it is the purpose of the current randomized clinical trial to test the hypothesis that immediate loading of a single mandibular midline implant with an overdenture will result in a comparable clinical outcome as using the standard protocol of delayed loading.

**Methods/design:**

This prospective nine-center randomized controlled clinical trial is still ongoing. The final patient will complete the trial in 2016. In total, 180 edentulous patients between 60 and 89 years with sufficient complete dentures will receive one median implant in the edentulous mandible, which will retain the existing complete denture using a ball attachment. Loading of the median implant is either immediately after implant placement (experimental group) or delayed by 3 months of submerged healing at second-stage surgery (control group). Follow-up of patients will be performed for 24 months after implant loading. The primary outcome measure is non-inferiority of implant success rate of the experimental group compared to the control group. The secondary outcome measures encompass clinical, technical and subjective variables. The study was funded by the Deutsche Forschungsgemeinschaft (German research foundation, KE 477/8-1).

**Discussion:**

This multi-center clinical trial will give information on the ability of a single median implant to retain a complete mandibular denture when immediately loaded. If viable, this treatment option will strongly improve everyday dental practice.

**Trial registration:**

The trial has been registered at Deutsches Register Klinischer Studien (German register of clinical trials) under DRKS-ID: DRKS00003730 since 23 August 2012. (http://www.germanctr.de).

## Background

Edentulism still occurs in elderly people in Germany and all over the world. In Germany, 30.5% of the population between 65 and 74 years are edentulous in the maxilla and/or mandible and 22.5% are completely edentulous [1]. Over the years there has been a strong consensus in dentistry that at least two implants are required to retain a complete denture in the edentulous mandible [2,3]. Indeed, two implants in the interforaminal area have high implant success rates and improve masticatory function [4-6]. In addition, a systematic review on the standard care for an edentulous mandible concluded that there is no strong evidence supporting a single standard of care for the edentulous mandible [7]. For elderly patients with a severely resorbed mandible, the use of implants is considered a medical necessity to retain dentures adequately and to reduce the resorption rate of the residual ridge in the anterior mandible. But as edentulism is often connected to having a low income [8], many patients cannot afford an implant therapy with two implants.

The concept of a single median implant in an edentulous mandible was introduced by Cordioli in 1993 [9] and the first 5-year results were published in 1997 with implant success rates of 100% [10]. A randomized clinical trial with 86 edentulous patients, compared mandibular overdentures retained by one or two implants [11]. Mandibular overdentures retained by a single median implant showed comparable satisfaction and maintenance time with lower component costs and treatment time than conventional two-implant overdentures during an observation time of 12 months. In these investigations, implants were left unloaded during healing. In 2010, Liddelow and Henry reported on a 100% implant-survival rate of immediately loaded implants after 36 months of observation when implants with oxidized surfaces were used [12]. Unfortunately, there was no control group with implants following a conventional healing protocol. However, Kronström *et al*. reported a failure rate of 17.6% after 12 months when immediate loading of a single median mandibular implant was applied [6]. So results of immediate loading of a single median mandibular implant retaining an overdenture are conflicting.

Therefore, the present study is designed to show that implant success is not compromised by immediate loading when a single median implant in the edentulous mandible is used to retain a complete mandibular denture. In addition it is the aim of this trial to show that the quality of life and chewing function can be improved earlier by immediate loading with less pain and discomfort for the patient.

### Objectives

This clinical trial will provide scientific evidence on the suitability of immediate loading of implants in an edentulous mandible. For health and economic reasons, a single median implant in the edentulous mandible is chosen as the most cost-effective treatment having a minimal number of implants. Approving immediate loading of a single dental implant in an edentulous mandible will have an enormous effect on the treatment of edentulous patients who have insufficient function of their complete dentures. As a result of this trial, a low-cost highly effective treatment modality might be established for a large group of edentulous patients in Germany.

## Methods/design

This study was designed as a prospective multi-center randomized controlled clinical trial. It will be conform to the CONSORT statement [13]. The study was designed according to Good Clinical Practice (GCP), the principles of the Declaration of Helsinki (2008) and standards for professional conduct.

A consensus for the study design, including patient treatment, materials used, length and frequency of follow-up visits, was achieved. A detailed study protocol exists. To ensure patient safety, an independent data safety and monitoring board has been installed. The study is monitored by an independent monitor to ensure GCP guidelines. The principal investigator approached eight additional departments of prosthetic dentistry in Germany to participate in the trial. One department refused to participate because of existing pressure of work. The study design was approved by the Ethics Commission of the University Hospital Schleswig-Holstein (processing number: AZ 138/12) as well as the Ethics Commissions of all the other participating centers: Ethics Commission of University Hospital Freiburg, Ethics Commission of Heidelberg University Hospital, Ethics Commission of RWTH Aachen University, Ethics Commission of Ulm University Hospital, Ethics Commission of University Hospital Hannover, Ethics Commission of Ernst-Moritz-Arndt University of Greifswald, Ethics Commission of the Dresden University of Technology and the Ethics Commission of the General Medical Council of the city of Hamburg.

### Participants

Patients wishing to participate in this clinical trial have to meet the following inclusion criteria:

– Provided written informed consent to participate in the trial.

– Edentulous male or female patient between 60 and 89 years.

– No contraindications for implantation.

– Sufficient bone in the anterior mandible to place an implant without augmentation procedures.

– Residual bone height is 11 to 20 mm (Class II or III according to McGarry *et al*. [14]) as the lowest vertical height of the mandible and the vertical bone height in the midline of the mandible is at least 13 mm.

– Despite technically acceptable complete dentures in the mandible and maxilla, the patient is unsatisfied with the retention and/or stability of the mandibular denture while the denture in the maxilla has adequate retention and stability.

– Existing dentures have been worn for at least 3 months to allow adaptation.

– Dentures must have a bilaterally balanced occlusal scheme.

Patients with any of the following conditions will be excluded:

– Edentulous patients aged 60 to 89 years with a contraindication for implantation in the mandible caused by systematic diseases or local bone deficits.

– Patients satisfied with the retention of their mandibular denture or who are unsatisfied with the retention and/or stability of their denture in the maxilla.

– Denture height between base and denture tooth central anterior less than 6 mm.

– Subjects with SCL-90, German version index T-scores of 70 or greater, or with two symptom scale scores of 70 or greater.

– Signs that the patient will be uncompliant and will not participate fully according to the test schedule.

– Participation in any former clinical trial should have been finished for more than 2 weeks.

### Study sites

Prosthetic departments of dental schools in Germany interested in clinical research were informed of the design and the aims of this study. Nine departments decided to participate. Each department designated an investigator responsible for patient examinations and follow-up visits and a clinician responsible for patient treatment (implant placement and second-stage surgery).

### Intervention

Two treatment groups are defined. For the experimental group, the median implant is immediately loaded after placement using a ball attachment and a matrix, which is integrated into the existing denture base intraorally. For the control group, the median implant is left unloaded and allowed to heal submerged for 3 months. The implant is loaded after second-stage surgery using a ball attachment and an intraorally placed matrix.

To avoid bias, randomization is conducted after implant placement and primary implant stability measurement by opening a sealed envelope. Neither the patient nor the study team knows the treatment group at the time of implant placement. Randomization is performed centrally by the trial statistician using block randomization with variable block size and an allocation ratio of 1:1. Stratification is according to the patient’s residual bone height (Class II or III according to McGarry *et al*. [14]) and the study center.

All interventions are conducted according to defined standard operating procedures. During the first calibration meeting, videos demonstrating the clinical und surgical procedures are shown. These are made available to all participating study centers. The first implant placement in each center is supervised by the treatment coordinator of the leading center. Experienced clinicians perform the surgical and prosthetic procedures.

### Outcomes

Implant success according to the modified success criteria of Albrektsson *et al*. [15] has been chosen as the primary outcome of this trial. Secondary outcomes are the earlier improvement in quality of life and chewing function tested using the German version of the oral health impact questionnaire [16] and objective masticatory performance [17].

It is hypothesized that patients with immediate loading will suffer less pain and discomfort during the intervention than patients with second-stage surgery as measured by a questionnaire with a visual analog scale. Prosthetic complications and maintenance interventions, such as adjustment or exchange of retention elements, fracture of the denture base, relining and so on, are recorded and should be comparable in both groups.

**Figure 1:**
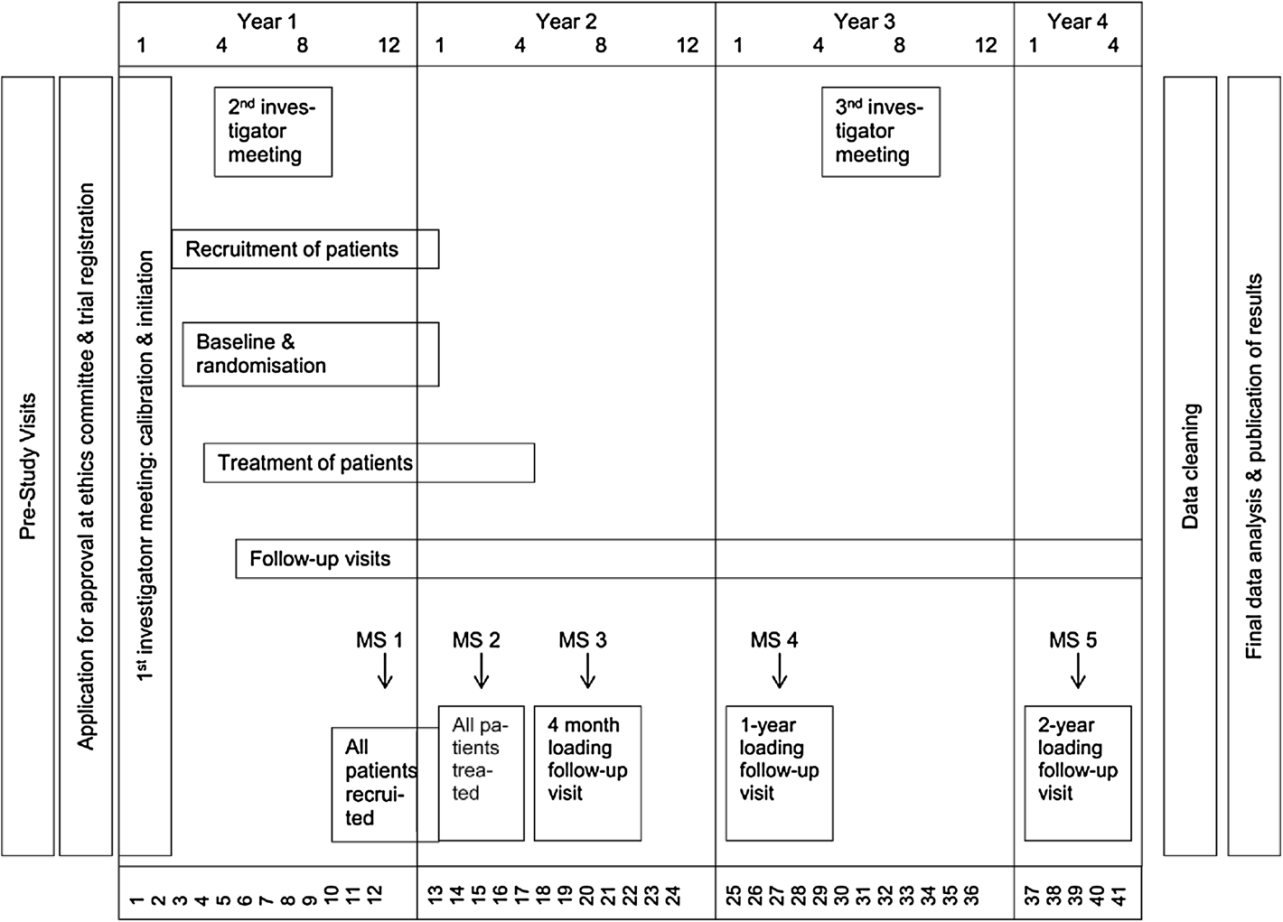
Organization chart of trial design.

Follow-up visits will occur at 1 month and 4, 12 and 24 months after implant loading. In addition, patients in the control group will be examined 1 month after implant placement (Figure 1).

### Stopping criteria

A patient will be removed from the trial if any of the following occur:

– Any complication during implantation.

– A minimum insertion torque of 30 Ncm and/or a minimum implant stability quotient of 60 is not achieved (the patient will not be randomized and will be treated as the control group).

– An allergic reaction to titanium.

– A serious adverse event related to the implantation.

– Any relevant deterioration in the health of the subject possibly affecting participation in the trial.

– Failure to comply with trial requirements.

– Withdrawal of consent.

### Sample size

The success rate of a single median implant after 2 years as the primary endpoint of the study is a binomial random variable and success probability is assumed to be 97% in the control group as well as in the experimental group. A maximum inferiority of 7% in the experimental group is regarded as clinically acceptable due to the benefits for these patients, i.e., avoiding the burden of a second procedure, having a stable denture directly after surgery and most probably reduced postoperative pain and discomfort, since the soft tissue of the surgical wound is not loaded with the denture during healing. Under these assumptions, a one-sided test of binomial parameters at a 2.5% significance level has a power of 80% to reveal the non-inferiority of the immediately loaded median implant if the sample size is 148 (74 per group). Taking into account a loss to follow-up rate of approximately 20%, a total of 180 patients (90 per group) was considered necessary [18]. It was assumed that the reasons for loss to follow-up would be random with respect to treatment assignment.

### Blinding

Due to the obvious visible differences in treatment, it is not possible to blind the investigator or the participating patient.

### Statistical methods

For the primary outcome, the measure for non-inferiority of the success rate of the experimental group compared to the control group will be tested by a one-sided equivalence test for the success parameter of the underlying binomial distributions. The significance level for the one-sided equivalence test is 2.5%. All patients that are randomized and followed up for 24 months as well as all patients with an implant failure will be statistically analyzed. The secondary endpoints (improvement of the chewing efficiency and improvement of the oral health related quality of life after the intervention) will be addressed by non-parametric tests for non-inferiority of the experimental group compared to the control group. Non-inferiority of prosthetic complications will be analyzed by one-sided equivalence tests.

In the case of an implant failure in the experimental group, the patient will be treated according to the protocol of the control group with a new implant. Those patients will be excluded from the trial before retreatment. If more than 20% of the implants fail in any group within the first 3 months after implant placement, the study will be terminated. This criterion will be checked every 6 months after the inclusion of the first patient.

### Recruitment

Recruitment of patients will be performed in two steps. Edentulous patients who have signed the informed consent will be screened according to the inclusion and exclusion criteria applying standardized examination forms and questionnaires including the SCL-90-index. Patients who otherwise meet the inclusion criteria but whose complete dentures are judged not to be technically acceptable will be referred for denture revision or new dentures. They will be offered the opportunity to be examined again after these improvements to the denture for possible inclusion in the trial if the dentures can be worn for at least 3 months before the recruitment period ends.

Patients meeting the inclusion criteria regarding denture status, denture satisfaction and SCL-90-index will undergo a radiographic examination (panoramic x-ray with reference marker) to determine whether the residual bone height of the mandible meets the inclusion criteria. To achieve consistent evaluation of the x-rays, the trial coordinator will assess all x-rays from each patient and perform the required measurements. For quality assurance, each patient’s documents will be sent pseudonymized to the treatment coordinator, who will make the final decision on inclusion in the study. If all inclusion criteria are met, patients will be included in the study.

## Discussion

This randomized clinical trial is designed to show the suitability of immediate loading of single median implants in an edentulous mandible. It is hypothesized that there will be non-inferiority of implant success for the experimental group compared to the control group.

Present clinical trials investigating a single midline implant in an edentulous mandible are mostly designed with a small number of patients [6,19,20]. Due to the sample size required for our clinical trial, a multi-center design is necessary. Taking into account a loss to follow-up rate of approximately 20%, a total of 180 patients (90 per group) was considered necessary. The loss to follow-up rate of 20% appears rather conservative but this rate also takes into account the exclusion of patients due to stopping criteria, e.g. compromised implant stability. In one study on immediate loading of a single mandibular implant, only 1 out of 17 implants (5.9%) did not achieve a minimum insertion torque of 30 Ncm [6]. In another study of immediate loading, only 2 out of 27 implants (7.4%) with oxidized surfaces did not achieve an insertion torque of 45 Ncm; however, the insertion torque actually obtained for these two implants was not reported [20]. From these studies, it is assumed that in our trial the number of patients excluded due to compromised implant stability and loss to follow-up will be less than 20%.

In a pilot study conducted at the University of Kiel, 11 patients were treated with one median implant in the mandible [21,22]. During this pilot trial, six implants followed a non-submerged healing protocol and were provided with a healing abutment and thus loaded moderately. No implant failed. The initial idea for the present clinical trial was to design a third treatment group as another experimental group with implants healing unsubmerged and moderately loaded over the healing abutment. However, for this three-arm study design, 354 patients would need to be allocated to the trial, as 282 patients would be needed for analysis. This number of patients would not have been feasible, especially in terms of finance. The idea of the present clinical trial is to compare two different healing protocols and not two different treatment options. The non-inferiority of one versus two implants in an edentulous mandible was shown by Walton *et al*. in 2009 [11]. A third treatment group with two implants in the interforaminal area was thus not considered necessary.

The defined inclusion criteria seem to be rather conservative, especially in terms of the residual bone height. We decided to include patients measured as McGarry Class II and III only. Patients with a residual bone height of 10 mm or less as the lowest vertical height of the mandible (Class IV) are excluded due to the risk of a rotational axis in the implant area and insufficient bone support of the mandibular overdenture in the posterior region. Patients with a residual bone height of 21 mm or greater as the lowest vertical height of the mandible (Class I) are excluded as well. It was expected that those patients’ quality of life related to oral health and prosthodontic satisfaction would already be at a rather high level and would not be significantly improved with an implant therapy requiring surgical intervention. It was estimated that 230 people assessed for eligibility would result in 180 people being allocated to the trial (an exclusion rate of 21.7%). So far, 19.1% of patients who have undergone screening have had to be excluded due to insufficient bone according to McGarry [14]. The residual bone height seems to be a major factor when considering edentulous patients for implant therapy. Psychological factors were of minor importance. Due to the high exclusion rate after screening during the first 8 months of recruitment, the recruitment period was extended until March 2014.

## Trial status

At the time of submission of this paper, patient recruitment is still ongoing. Patients will be enrolled in the trial until the end of March 2014. Recruitment will be stopped automatically as soon as 180 patients have been included.

## Abbreviations

GCP: good clinical practice.

## Competing interests

The authors declare that they have no competing interests.

## Authors’ contributions

NP conceived of and designed the study, collected and analyzed the data, wrote the manuscript and gave final approval for the manuscript. MK is the principal investigator, conceived of and designed the study, collected and analyzed the data, wrote the manuscript and gave final approval for the manuscript. RL conceived of and designed the study, collected and analyzed the data, provided a critical revision and gave final approval for the manuscript. EF conceived of and designed the study, undertook statistical analysis, provided a critical revision and gave final approval for the manuscript. MB, SK, TK, NvM, AvS, TM and MR collected and analyzed the data, provided a critical revision and gave final approval for the manuscript. All authors read and approved the final manuscript.
